# ‘Second primary breast cancers following an initial diagnosis of cancer in one breast: a methodological issue’

**DOI:** 10.1038/sj.bjc.6604718

**Published:** 2008-10-14

**Authors:** D Robinson

**Affiliations:** 1 King's College London, Thames Cancer Registry, Capital House, 42 Weston Street, London, SE1 3QD, UK

**Sir**,

A large number of studies have been published investigating the occurrence of second primary cancers following the diagnosis of an ‘index’ cancer. The standard method of analysis is to compare the observed numbers of subsequent cancers with the numbers expected on the basis of cancer incidence rates in the general population (accounting for age, sex and time period). To do this, the number of ‘person-years at risk’ is calculated, with the ‘at risk’ period for each individual starting at the date of diagnosis of the index cancer and terminating at the study cutoff date, the date of death or loss to follow-up, or the date of diagnosis of the subsequent cancer of interest. If the treatment for the index cancer entails removal of the organ in which this occurred, then it seems sensible when investigating the occurrence of second cancers at the same anatomical site to terminate the ‘at-risk’ period at the point when the removal occurred, as a subsequent primary cancer cannot arise in an organ that no longer exists.

For example, a woman with breast cancer who undergoes a total mastectomy is no longer at risk for subsequent ipsilateral breast cancer, but she does remain at risk for subsequent contralateral breast cancer. Consequently, ipsilateral and contralateral cancers should be treated differently in the calculation of time at risk. For contralateral breast cancer, the standard method of calculating time at risk applies. However, for ipsilateral breast cancer the ‘at-risk’ period should be truncated at the date of mastectomy if this has been performed. Expected numbers of subsequent ipsilateral and contralateral breast cancers are then calculated by multiplying the ‘at-risk’ period by half the standard breast cancer incidence rate, and the two added together to give the total expected number of subsequent breast cancers.

Failure to correct for mastectomies leads to underestimation of the standardised incidence ratios (SIRs) for ipsilateral tumours. This is because, without the correction, mastectomy cases are considered to be at risk until the cutoff date at the end of the study. As a result, person-years at risk (and hence estimated expected numbers) are inflated. An example of this effect is given in Table 1, which shows the observed numbers of subsequent invasive breast cancers in a series of 13 269 women diagnosed with breast carcinoma *in situ* (Robinson *et al*, 2008), together with the expected numbers calculated using the standard methodology (‘no correction’) and after applying the correction for mastectomies as described above (‘correction’). Without the correction, the SIR for ipsilateral breast cancer is lower than for contralateral tumours. After applying the correction, this is reversed. It seems intuitively more likely that a second breast cancer would develop in the breast where *in situ* disease had been detected.

This problem has been recognised for some time. Franceschi (1997) pointed out in an editorial that it is ‘worth bearing in mind that the computation of the expected number of breast cancers in prospective studies of women whose breasts have been partly removed is open to discussion’. Levi *et al* (1998) concurred, adding that expected values are ‘likely to be overestimated’. However, this effect is rarely taken into account. Rawal *et al* (2005) analysed ipsilateral and contralateral breast cancers separately, but it appears that mastectomies were not accounted for. Similarly, Soerjomataram *et al* (2006) calculated expected numbers and SIRs for breast cancer following breast carcinoma *in situ* separately for ipsilateral and contralateral tumours, but it is not clear whether they allowed for mastectomies. Moreover, they applied the overall incidence rates from the general population to each group (rather than half-rates), thus leading to expected numbers which were twice, and SIRs which were half, the true values. This issue of applying half-rates was addressed by Peto (1987) in the context of calculating expected numbers of contralateral breast cancers.

Similar arguments would apply in other situations involving organ removal – for example, hysterectomies should be taken into account when looking at subsequent occurrences of cervical or uterine cancers. These issues should be borne in mind when investigating the occurrence of second cancers.

## Figures and Tables

**Table 1:** Effect of mastectomy ‘correction’ on estimated standardised incidence ratios (SIRs)

**Side**	**Observed**	**Expected**	**SIR**
Contralateral	286	165.99	1.72
			
*Ipsilateral*			
No correction	226	167.08	1.35
Correction	226	95.45	2.37
			
*Total*			
No correction	512	333.07	1.54
Correction	512	261.44	1.96
